# Thoracic Ultrasound Utility in Pulmonary Pathologies Following Blunt Chest Trauma: A Cross-Sectional Study From Barcelona, Venezuela

**DOI:** 10.7759/cureus.57520

**Published:** 2024-04-03

**Authors:** Jennifer Uzcategui-Gutierrez, Yeisson Rivero-Moreno, Georcimar Mendez-Meneses, Yoalkris E Salcedo, Wilson Garcia-Cazorla, Laila Tarabey-Yunis, Emiliana Garcia-Sánchez, Debbye Machado-Paled, Cesar Estrella-Gaibor, Tamara Rodriguez-Rugel, Luis Mejías-Caraballo

**Affiliations:** 1 Department of Surgery, Universidad de Oriente, Núcleo Anzoátegui, Barcelona, VEN; 2 Department of Surgery, Universidad Iberoamericana, Santo Domingo, DOM; 3 Department of Surgery, Universidad de Cuenca, Cuenca, ECU; 4 Department of Surgery, Universidad Centroccidental Lisandro Alvarado, Lara, VEN; 5 Department of Surgery, Hospital Regional Dr Franco Ravera Zunino, Rancagua, CHL; 6 Department of Surgery, Universidad Católica de Honduras, Tegucigalpa, HND; 7 Department of Surgery, Ministerio de Salud Pública, Hospital Esmeraldas sur Delfina Torres de Concha, Quito, ECU; 8 Department of Surgery, Universidad Católica de Santiago de Guayaquil, Guayaquil, ECU

**Keywords:** hemothorax, pneumothorax, chest radiography, blunt chest trauma, thoracic ultrasound

## Abstract

Background

The thoracic ultrasound (TUS) is a monitoring tool that has gained worldwide popularity in various scenarios, offering the opportunity for dynamic, bedside evaluations. Recent studies indicate that the use of TUS enables the diagnosis of pathologies resulting from blunt chest trauma (BCT), yielding favorable outcomes. This study aimed to compare the utility of TUS versus chest radiography (CXR) in diagnosing pulmonary pathologies resulting from closed-chest traumas.

Methodology

A prospective cross-sectional study was conducted with a sample of 58 patients diagnosed with BCT who sought emergency care at the “Dr. Luis Razetti” University Hospital in Barcelona, Venezuela, from November 2023 to January 2024.

Results

Of the patients, 75.9% (n = 44) were male, with an average age of 37.8 years (standard deviation = 18.4 years). Injuries were reported in 8.6% (n = 5) of the patients, including 60% (n = 3) pneumothorax and 40% (n = 2) hemothorax. Ultrasound results coincided with CXR in 94.8% (n = 55) of the cases, with a Cohen’s kappa coefficient of 0.9 (95% confidence interval (CI) = 0.642-1.0). TUS demonstrated higher sensitivity than CXR (100% vs. 60%) for detecting hemothorax and pneumothorax in patients with BCT, with an area under the receiver operating characteristic curve of 0.991 (95% CI = 0.968-1.013).

Conclusions

BCT predominantly occurred in young males, resulting primarily in pneumothorax and hemothorax lesions, detectable with higher sensitivity through TUS compared to CXR. The use of TUS should be considered an essential component of the initial assessment for individuals with BCT.

## Introduction

Thoracic ultrasound (TUS) is a monitoring tool that has gained global prominence in various settings, providing significant support in medical practice by offering the opportunity for dynamic, bedside assessments. It holds distinct advantages over other imaging studies, including on-the-spot diagnosis at the patient’s bedside, absence of radiation, cost-effectiveness, accessibility, portability, reproducibility, safety for both the patient and the healthcare professional or technician performing it, and a reduction in complications during interventional procedures [1].

In the ultrasound assessment of the thoracic wall, visualization of superficial structures, such as the skin, intercostal musculature, costal arches, as well as the pleura and lung parenchyma, is possible [2]. Essential to this assessment is the visualization of the pleura, appearing as a hyperechoic line that moves in accordance with respiration, known as the “lung sliding” sign [1,2]. Normal lung visualization results in the appearance of artifacts as the air-filled lung may seem invisible on ultrasound, preventing the acquisition of any image in a healthy lung. Conversely, if the lung parenchyma is replaced by tissue, fluid, or consolidations, a valuable image can be obtained to identify the presence or absence of pathology. Gravity causes fluid to accumulate at the bases, while air is present at the pulmonary apices, prompting a targeted ultrasound search based on the suspected pathology [3-7].

The imaging studies used to confirm the diagnosis in patients with trauma include chest radiography (CXR), TUS, and chest computed tomography (CT). CT is considered the gold standard for diagnosing pathologies in chest trauma; however, recent studies suggest that the systematic use of TUS allows for a diagnosis with sensitivity and specificity similar to that of CT [8].

TUS is more accurate than CXR in diagnosing pneumothorax in trauma patients presenting to the emergency department. This can lead to more timely treatment through thoracotomy, reducing complications associated with pneumothorax and improving outcomes. The risk of incorrectly diagnosing traumatic pneumothorax through TUS is low [8].

Overall, trauma is the third leading cause of death in patients under 40 years old. Thoracic trauma is considered responsible for 20-35% of these fatalities. The majority of traumatic injuries to the chest are caused by car collisions, followed by penetrating wounds. Clinically, thoracic trauma is classified into contusive or closed injuries and penetrating or open injuries. Among the various injuries are rib fractures, clavicle fractures, sternum fractures, scapula fractures, unstable chest, cardiopulmonary contusions, pneumothorax, hemothorax, vascular injuries, and damage to upper digestive organs, among others. The initial approach must strictly follow the guidelines for the care of polytrauma patients. Blunt trauma is a common presentation of thoracic trauma. The diagnostic approach for blunt chest trauma (BCT) includes multiple diagnostic aids, including TUS, CXR, and chest CT [9-11].

The objective of this study was to compare the utility of TUS against CXR in the diagnosis of pulmonary pathologies resulting from BCT.

## Materials and methods

A prospective cross-sectional study was conducted among patients who presented to the emergency department of general surgery with BCT at the “Dr. Luis Razetti” University Hospital in Barcelona, Venezuela, between November 2023 and January 2024.

Patients over 12 years old diagnosed with BCT resulting from direct trauma, crushing injuries, run-over incidents, or traffic accidents who were admitted during the study period and underwent both TUS and CXR as part of their initial evaluation were included. Pregnant patients and those with incomplete data were excluded. TUS was performed by the attending physician on duty with a diploma in pulmonary ultrasound, and either in-house or external posteroanterior CXR was conducted for the included patients. Additionally, some patients underwent chest CT scans to confirm certain findings. All imaging studies were conducted within the first 48 hours of patient admission.

Information regarding age, gender, TUS, CXR, and chest CT scan results for select patients was extracted. Data were obtained from clinical records and official reports of the imaging studies. The final sample size was determined through convenience sampling based on patients who met the inclusion criteria.

Data were expressed as mean and standard deviation (SD) or as percentages for quantitative and qualitative variables, respectively. The exact Mid-P test was used to assess the association between qualitative variables, and the Student’s t-test was used to evaluate differences between averages. Cohen’s kappa coefficient was employed to assess the concordance between the results of the diagnostic tests used, with interpretation based on McHugh et al. criteria shown in Table 1 [12].

**Table 1:** Interpretation of Cohen’s kappa [12].

Value of kappa	Level of agreement
0–0.20	None
0.21–0.39	Minimal
0.40–0.59	Weak
0.60–0.79	Moderate
0.80–0.90	Strong
Above 0.90	Almost perfect

Additionally, a receiver operating characteristic (ROC) curve analysis was conducted to evaluate the efficacy of TUS in detecting injuries such as hemothorax and pneumothorax. A p-value <0.05 was considered statistically significant. Statistical analysis was performed using SPSS version 29 software (IBM Corp., Armonk, NY, USA) and the online tool OpenEpi version 3.01. The structure of this study adhered to the STROBE guidelines for cross-sectional studies [13].

## Results

During the study period, a total of 58 patients were included. Of these, 75.9% (n = 44) were male, and the average age was recorded as 37.8 years with an SD of 18.4. The age range of the studied sample was 12 to 83 years, with the most common age group being 20 to 44 years, representing 48.3% (n = 28) of the patients. Details of the age and gender of the studied patients are presented in Table 2. There was no statistically significant difference between females and males regarding age group (p = 0.566) or average age (p = 0.203).

**Table 2:** Demographic characteristics of patients with blunt chest trauma.

Demographic characteristics	N (%)
Sex	
Male	44 (75.9)
Female	14 (24.1)
Age group	
12–19 years	10 (17.2)
20–44 years	28 (48.3)
45–59 years	12 (20.7)
>60 years	8 (13.8)

In addition to TUS and CXR, chest CT scans were successfully performed for 6.9% (n = 4) of the patients to confirm or rule out the presence of injuries. A definitive diagnosis of BCT injuries was considered when two out of the three diagnostic methods used (TUS, CXR, or chest CT) concurred on the diagnosis. Based on this criterion, pathological findings were reported in 8.6% (n = 5). Among the five patients with confirmed injuries in different imaging tests, 60% (n = 3) presented pneumothorax, and 40% (n = 2) presented hemothorax. Additionally, two patients concurrently exhibited rib fractures on the CXR. The details of the injuries found for each type of imaging study are outlined in Table 3.

**Table 3:** Distribution of injuries in patients with blunt chest trauma. TUS = thoracic ultrasound; CXR = chest X-ray; CCT = chest computed tomography scan

Injuries, n (%)	TUS (n = 58)	CXR (n = 58)	CCT (n = 4)
Normal	52 (89.6)	53 (91.4)	1 (25)
Hemothorax	2 (3.4)	1 (1.8)	2 (50)
Pneumothorax	4 (7)	2 (3.4)	1 (25)
Rib fracture	-	2 (3.4)	-

The frequency of hemothorax and pneumothorax injuries was evaluated based on the demographic characteristics (age and sex) of the studied patients. In the group of patients with detected injuries in the TUS, the average age was higher compared to patients who did not have injuries in this study (p = 0.014). Details are provided in Table 4 and Table 5.

**Table 4:** Relationship between demographic characteristics and injuries found in CXR of patients with blunt chest trauma. ^a^ = Qualitative variables are expressed as number (%) and quantitative variables as mean and standard deviation. ^*^ = Student's t-test. ^†^ = Mid-P exact test. Statistically significant values are in bold. CXR = chest X-ray

Demographic characteristics^a^	Without injuries in the CXR (n = 55)	With injuries in the CXR (n = 3)	P-values
Age	36.3 (17.8)	57 (21.5)	0.057*
Gender	-		
Female	13 (23.6)	1 (33.3)	0.712†
Male	42 (76.4)	2 (66.7)	

**Table 5:** Relationship between Demographic Characteristics and Injuries Found in TUS of Patients with Blunt Chest Trauma. ^a^ = Qualitative variables are expressed as number (%) and quantitative variables as mean and standard deviation. ^*^ = Student's t-test. ^†^ = Mid-P exact test. Statistically significant values are in bold. Statistically significant values are in bold. TUS = thoracic ultrasound

Demographic characteristics^a^	Without injuries in the TUS (n = 52)	With injuries in the TUS (n = 6)	P-values
Age	35.4 (17.8)	54.6 (14.6)	0.014*
Gender	-		
Female	13 (25)	1 (16.7)	0.724†
Male	39 (75)	5 (83.3)	

In 94.8% (n = 55) of cases, the results of TUS coincided with the CXR. In three patients, findings did not align, as TUS detected two pneumothoraxes and one hemothorax that were not visualized on the CXR. However, in two out of these three cases, these ultrasound findings were later confirmed by chest CT scans. Overall, the level of agreement between TUS results and the diagnosis of injuries based on CXR and chest CT was high, with a Cohen’s kappa coefficient of 0.9 (95% confidence interval (CI) = 0.642-1.0).

The sensitivity and specificity of TUS and CXR were evaluated and compared (Table 5) in detecting pneumothorax and hemothorax injuries. TUS exhibited higher sensitivity than CXR (100% vs. 60%) in detecting hemothorax and pneumothorax injuries in patients with BCT.

**Table 6:** Evaluation of sensitivity and specificity of TUS and CXR in patients with blunt thoracic trauma. TUS = thoracic ultrasound; CXR = chest X-ray; PPV = positive predictive value; NPV = negative predictive value

-	Patients with injuries	Patient without injuries	Total
TUS with injuries	5	1	PPV: 83.3%
TUS without injuries	0	52	NPV: 100%
-	Sensitivity: 100%	Specificity: 98.1%	-
CXR with injuries	3	0	PPV: 100%
CXR without injuries	2	53	NPV: 96.4%
-	Sensitivity: 60%	Specificity: 100%	-

**Figure 1:**
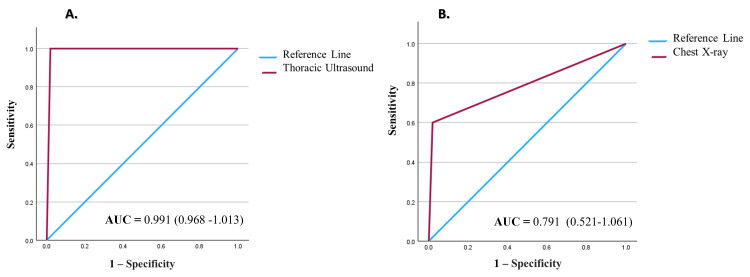
Receiver operating characteristic (ROC) curve for the prediction of hemothorax and pneumothorax after blunt chest trauma. AUC = area under the curve

The area under the ROC curve for TUS was 0.991 (95% CI = 0.968-1.013), indicating a robust discriminative capacity for detecting hemothorax and pneumothorax following BCT. In contrast, the area under the ROC curve for chest X-ray was 0.791 (95% CI = 0.521-1.061). Figure 1a and Figure 1b illustrate both ROC curves.

## Discussion

A prospective study was conducted to assess the use of TUS compared to CXR in diagnosing pathologies resulting from BCT. The study found that ultrasound demonstrated higher sensitivity than X-ray in detecting injuries such as hemothorax and pneumothorax.

The male gender was the most frequently affected, and the most common age group was 22 to 44 years. Similar results have been reported by other studies related to BCT, such as the study by Mogollón-Guzmán et al. in 2019, where they analyzed the characteristics of 268 Ecuadorian patients with chest trauma. They reported that the majority of patients (57.3%) had BCT, with males being the most frequent at 72.4% of cases and an average age of 38 years [14].

The study by Gonçalves et al. in 2023, involving 100 patients who were victims of thoracic trauma treated at an emergency hospital in Brazil, similarly reported that 85% of those affected were men, with an average age of 39.3 years. The most common age range was between 30 and 39 years [15].

The higher frequency of men in this type of pathology is attributed to their greater involvement in risky activities, including high-speed vehicles, motorcycles, violence, and falls from heights due to occupational factors [16].

When we evaluated the relationship between age, gender, and the frequency of detected injuries, patients with hemothorax and pneumothorax injuries had a higher average age. This is expected considering the predisposition to more injuries in older patients. Increasing age is an independent risk factor for complications after traumatic injury. Elderly patients (defined as those over 65 years old) have morbidity and mortality four times higher compared to younger patients with the same severity score, especially after thoracic or cranioencephalic trauma [17]. Furthermore, age greater than 60 years is a risk factor for mortality after thoracic trauma [18], and it has even been reported as a risk factor in those over 50 years old [19].

In this study, pneumothorax was the most frequently reported injury, followed by hemothorax. Similar results have been found in different studies, such as the one by Araujo et al. in 2017, which evaluated a total of 200 patients with thoracic trauma in a hospital in Maracaibo, Venezuela. They reported pneumothorax as the main injury in 72% of patients with BCT, followed by hemothorax in 28% [20]. Satorre-Rocha et al. in 2019 reported similar findings in 102 patients with thoracic trauma treated in a hospital in Havana, Cuba [21]. Pneumothorax occurs due to the “paper bag effect” (lung rupture when closing the glottis before impact) and is a result of tearing or shearing injuries in frontal and lateral impacts, the primary mechanisms of injury in BCT. Chest wall injuries can lead to rib fractures with or without an unstable chest, cardiac contusion, arrhythmias, pulmonary contusion, and pneumothorax [22].

Currently, the most widely used method for evaluating patients with BCT is CT due to its higher sensitivity compared to other techniques such as CXR, especially within the first six hours after trauma [23]. Therefore, it has been established as the gold standard for imaging evaluation of thoracic trauma and trauma in general, and it should be performed whenever possible in patients with this pathology [24]. However, in the sample of patients studied, this study was not available to precisely define potential underlying injuries.

Ultrasound is a valuable tool in the assessment and management of pleural pathology and has been increasingly used in recent years for the initial evaluation of traumatized patients. It is an imaging technique that provides high sensitivity, specificity, and accuracy, making it possible to detect small pleural effusions associated with chest trauma even before their identification on X-rays, allowing for early interventions. It is a cost-effective technique, requires less infrastructure, is versatile, and can be easily moved to assess critically ill patients in intensive care units, including those under mechanical ventilation [25]. The superior performance of ultrasound over CT in detecting retained hemothorax is explained by the fact that ultrasound provides better visualization of pleural fluid characteristics. It also evaluates the presence, thickness, and mobility of septa within the pleural space. It should also be considered that the density of subacute or chronic intrapleural clots is lower compared to the density of the clot in its acute phase. This makes the increase in density in the pleural space not evident when evaluating it by CT [25].

In our study, the injuries detected by TUS were confirmed by chest CT, even in cases that did not coincide with CXR, demonstrating an even higher sensitivity (100% vs. 60%). In fact, when comparing CXR with CT for the detection of post-traumatic residual hemothorax, it is insufficient to select patients who should undergo surgery, as, in a third of cases, the findings were overestimated; this is according to the study by Zinck et al. [26]. Additionally, in the study by Jivani et al. in 2023, where they evaluated 64 patients with closed thoracic trauma, CXR showed a sensitivity of 28.6% for the diagnosis of pneumothorax and pneumomediastinum [27], similar to the findings reported by Moya et al., who showed that almost 10-50% of pneumothoraces from closed thoracic trauma are not visualized on CXR [28]. In our study, the high sensitivity reported with the use of TUS compared to conventional CXR demonstrates the clinical relevance of its implementation. This is because patients identified through TUS can be promptly managed medically or surgically as necessary. This ultimately suggests that in the context of injuries in patients with closed chest trauma, TUS may be superior to CXR.

On the other hand, various international studies have reported the performance of TUS compared to CXR or chest CT in detecting injuries from closed thoracic trauma. For instance, the study by Suárez-Poveda et al. in 2012, where they investigated 68 patients with suspected hemothorax, demonstrated that TUS had better diagnostic performance than contrast-enhanced CT in these patients, with a sensitivity of 72.3% and specificity of 95.24% [25].

In the study by Vafaei et al. in 2016, analyzing the results of 152 patients with thoracic trauma, it was reported that ultrasound for pneumothorax detection had a sensitivity/specificity of 83.6%/97.9%, compared to CXR with 67.3%/92.7%. For hemothorax detection, ultrasound showed a sensitivity/specificity of 75.9%/95.9% compared to CXR with 58.6%/95.1% [29]. Other studies have also demonstrated the superiority of ultrasound over CXR in these patients [30]. The superiority of ultrasound is due to its high sensitivity in detecting fluids and gases, making it effective in identifying the presence of fluid (such as blood in the case of hemothorax) or air (as in the case of pneumothorax) in the thoracic cavity. X-ray, on the other hand, may have limitations in detecting these substances, especially in the early stages. Additionally, ultrasound provides real-time images, allowing the physician to dynamically visualize the region in question and observe any movement, change, or fluctuation in the collection of fluid or air. This can be crucial for assessing the severity of the injury and guiding drainage procedures if necessary.

In Venezuela, there have been no published studies evaluating the role of ultrasound in the diagnostic management of patients with thoracic trauma. However, in Latin America, authors such as Brismat-Remedios et al. in 2021 reported that in 1,052 patients, extended TUS achieved a sensitivity of 95.24% and specificity of 99.88% in diagnosing thoracic injuries [31].

The conclusions drawn from this research may be limited by the low number of patients with detected lesions in the imaging studies, affecting the accuracy of results in terms of the relationship between variables, such as age concerning lesions in TUS, or the calculation of sensitivity and specificity of the diagnostic studies used. The lack of chest CT studies in most patients limited a definitive and standardized diagnosis with a high degree of certainty for the studied pathologies. Similar to other studies involving ultrasound in different anatomical regions, the results of this study could be influenced by operator bias during the TUS examinations. However, it is important to highlight that this study is the first to evaluate the use of TUS compared to other diagnostic tools for patients with BCT in Venezuela, demonstrating that this readily available tool may be even more useful than conventional imaging studies in most cases.

## Conclusions

BCT is one of the most common forms of thoracic trauma, primarily occurring in young men. It often leads to injuries such as pneumothorax and hemothorax, which can be detected through various imaging techniques, including CXR, TUS, and chest CT, with the latter being the gold standard for evaluating these patients. However, this valuable diagnostic tool is not always available in public healthcare centers in the country. Therefore, the use of TUS could play a crucial role in detecting pleuropulmonary pathologies, as it has demonstrated higher sensitivity than CXR for detecting such injuries. The use of TUS should be considered an indispensable part of the initial assessment of individuals with chest trauma.
